# An Unusual Case of Right Anterior Descending Coronary Artery

**DOI:** 10.7759/cureus.48090

**Published:** 2023-11-01

**Authors:** Dibyasundar Mahanta, Shilpa Vinayak Gadade, Deepak Kumar Parhi, Debasish Das

**Affiliations:** 1 Department of Cardiology, SUM Hospital, Bhubaneswar, IND; 2 Department of Cardiology, All India Institute of Medical Sciences, Bhubaneswar, IND

**Keywords:** right coronary artery, right anterior descending coronary artery, coronary sinus, left anterior descending coronary artery, anomalous

## Abstract

The left anterior descending coronary artery (LAD) arises from the left coronary sinus about 10-12 mm above the annular plane and traverses down the interventricular groove. With deep septal and diagonal branches, it supplies the left side of the heart. Here, we describe an extremely rare case of anomalous origin of the LAD from the right coronary artery, which courses epicardially over the right side of the heart with its ramifying branches, which can be described as the "right anterior descending coronary artery (RAD)."

## Introduction

Coronary anomalies are extremely rare to be encountered in routine interventional practice. The true incidence of coronary anomalies during invasive coronary angiography is estimated to be around 1% [1-3]. Coronary anomalies increase across complex congenital heart diseases such as tetralogy of Fallot, truncus arteriosus, and D-transposition of great arteries (D-TGA). The most common coronary anomaly encountered in routine clinical practice is the anomalous origin of the left circumflex coronary artery from the right coronary artery (RCA) or right coronary sinus. We describe here an extremely rare coronary anomaly where the left anterior descending coronary artery (LAD) was arising from the RCA and was traversing along the right anterior surface of the heart with ramifying branches, which can be described as the right anterior descending coronary artery (RAD). The anomalous coronary artery in the terminal part turned towards the left side to supply the left side of the heart. The ramifying branches of the RAD were supplying the right ventricular anterior myocardium. The patient had atypical chest pain secondary to relative coronary ischemia due to non-perfused LAD territory, as LAD was not following its natural course. 

## Case presentation

A 22-year-old obese male, daily laborer, and smoker presented to the cardiology outpatient department (OPD) with exertional angina for the last six months without any past history of palpitation, presyncope, or syncope. During the presentation, he had a heart rate of 84 beats per minute with a blood pressure of 130/80 mmHg in the right arm supine position. His cardiovascular system examination was within normal limits. The baseline ECG revealed no ischemic ST-T changes, and he had no regional wall motion abnormality with preserved left ventricular systolic function (ejection fraction (EF)=65%) in echocardiography. He was not able to perform the treadmill exercise test, could not achieve the age-predicted target heart rate, and was able to perform only six metabolic equivalents (METS) as he had profound diaphoresis with shortness of breath at six METS only. He was subjected to an invasive coronary angiogram in order to exclude congenital coronary anomalies, including myocardial bridging. CT coronary angiography could not be done as he had profound anxiety and tachycardia prior to the procedure.

**Figure 1:**
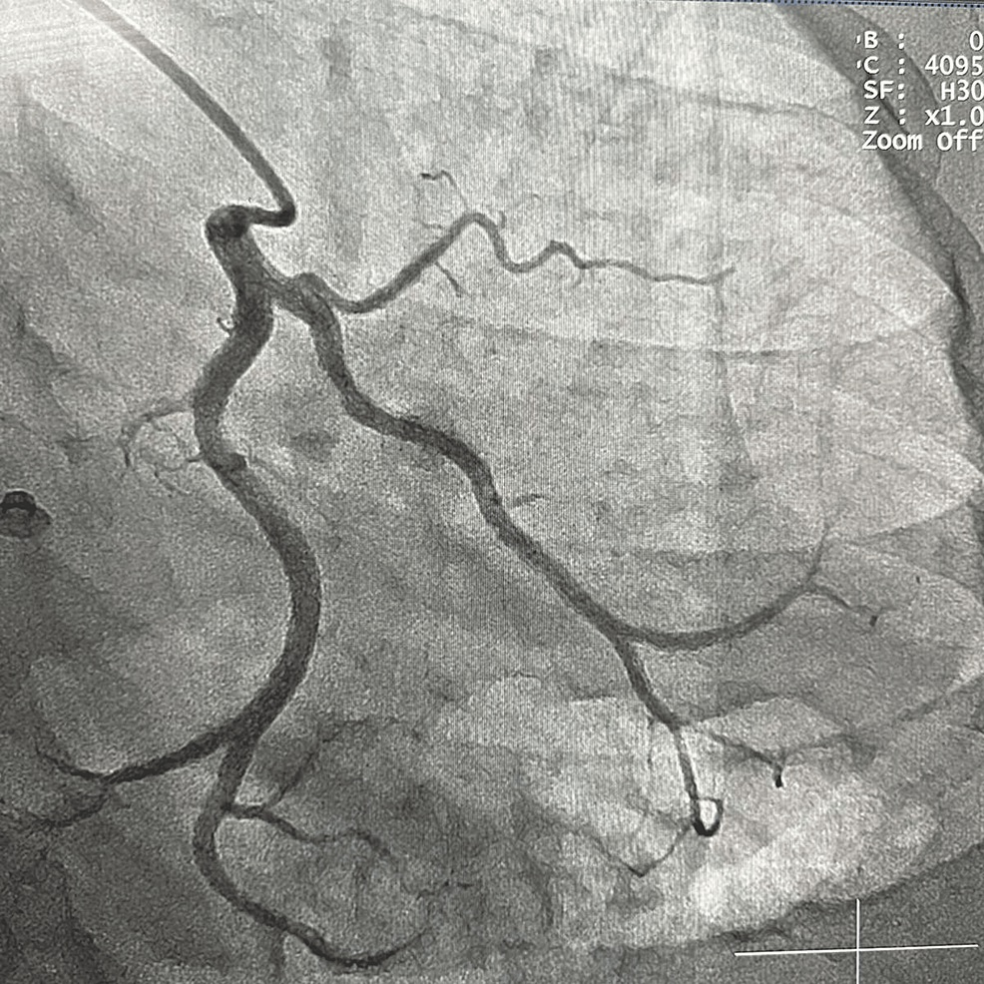
Absence of origin of LAD from the left coronary sinus. LAD: left anterior descending coronary artery.

**Figure 2:**
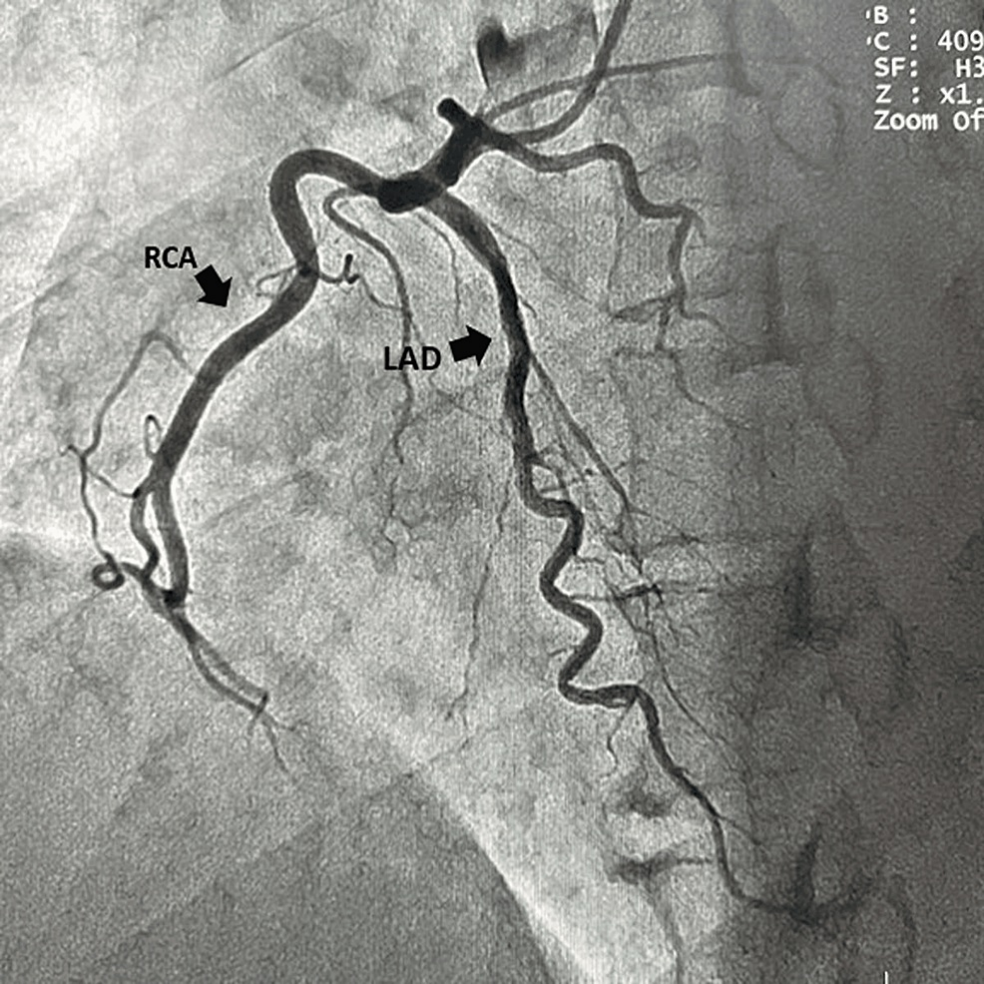
Origin of LAD from the RCA with ramifying branches of LAD on right ventricular myocardium. LAD: left anterior descending coronary artery; RCA: right coronary artery.

A right transradial coronary angiogram was performed, and a left coronary sinus injection revealed the only origin of the left circumflex coronary artery (LCX) from the left coronary sinus with avascular left anterior descending artery territory (Figure 1). Given the absence of a LAD, it was presumed that LAD might originate from the right coronary sinus. Injection of the RCA revealed that the LAD originated from the proximal part of the RCA and traversed over the right anterior surface of the heart, nonetheless to be described as "right anterior descending coronary artery" (Figure 2). Interestingly, this anomalous LAD gave rise to the septal from its left side as it traversed toward the left to supply the interventricular septum (IVS). Interestingly this anomalous coronary artery did not give rise to any diagonals, and the first obtuse marginal branch (OM1) of the LCX was large enough to compensate for the absence of the diagonal branches. The first branch of the OM1 was supplying the diagonal branch territory (Figure 1). This anomalous coronary artery was coursing around the apex of the heart to depict the classic "Moustache sign" of the LAD.

Our case presents an extremely rare description of the anomalous LAD originating from the proximal part of the RCA and completely coursing over the right anterior surface of the heart with the peculiar origin of the septal branches from its left side, nonetheless to be called "right anterior descending coronary artery (RAD)." The young boy had exertional angina, which may be secondary to a large area of avascular myocardium, i.e., absence of vascular arborization in LAD territory. 

## Discussion

The incidence of coronary anomalies during routine echocardiography is 0.17% [4], and the incidence of coronary anomalies during invasive coronary angiography is estimated to be around 1.07% [5]. We describe an extremely rare congenital coronary anomaly, i.e., the anomalous origin of the LAD from the RCA, in a young boy with exertional angina. Detection of anomalous coronary arteries at a very young age is of paramount importance, as these are associated with a high risk of angina, palpitation, syncope, and sudden cardiac death [5]. Anomalous coronary artery traversing between the aorta and pulmonary artery carries the highest risk of sudden cardiac death as it is more prone to get systolic compression between two great arteries during tachycardia in exertion and emotional stress [6].

The most interesting aspect in our case was that the anomalous coronary artery completely traversed over the right anterior surface of the heart, for which it was not providing any diagonal or septal branches and it was giving rise to the septals only in its terminal part when it was turning to the left side to supply the IVS. As it was almost coursing over the right anterior surface of the heart, it can be described as the RAD. In the normal heart, the septals originate from the right side of the LAD, and the diagonals originate from the left side of the LAD in anteroposterior cranial view (AP cranial), but in the index case, septals originated from the left side as the anomalous artery was completely coursing over the right anterior surface of the heart. There was no atherosclerotic obstruction in any of the coronaries, and the anomalous coronary artery did not reveal any inter-arterial course, which is a predictor of sudden cardiac death in this young person. The patient had a large area of avascular myocardium, which may be the reason for exertional angina in the absence of flow-limiting atherosclerotic obstruction [7]. Large areas of avascular myocardium also predispose to coronary ischemia as exemplified by our index case.

Although CT coronary angiography is the gold standard for delineating the three-dimensional course of the anomalous coronary artery, it could not be accomplished as the patient had profound tachycardia prior to the procedure. Routine CT coronary angiography usually requires a slow resting heart rate of 60-80 beats per minute for better acquisition of the cine frame images. The anomalous coronary artery is an important cause of myocardial ischemia in the young, and absent arborization in any major vascular territory always prompts search for the same in young persons with atypical chest pain or exertional angina. 

## Conclusions

Our case is an extremely rare case of anomalous LAD arising from the proximal part of the RCA, completely coursing over the right anterior surface of the heart and giving rise to the septals from its left side in its terminal part, nonetheless to be described as "right anterior descending coronary artery (RAD)." An anomalous coronary artery with non-arborized major vascular territory can be an attributing factor to angina in the young. 
